# An Innovative Metal Ions Sensitive “Test Paper” Based on Virgin Nanoporous Silicon Wafer: Highly Selective to Copper(II)

**DOI:** 10.1038/srep36654

**Published:** 2016-11-08

**Authors:** Shaoyuan Li, Xiuhua Chen, Wenhui Ma, Zhao Ding, Cong Zhang, Zhengjie Chen, Xiao He, Yudong Shang, Yuxin Zou

**Affiliations:** 1 State Key Laboratory of Complex Nonferrous Metal Resources Clean Utilization/National Engineering Laboratory for Vacuum Metallurgy, Kunming University of Science and Technology, Kunming 650093, China; 2 Faculty of Physical Science and Technology, Yunnan University, Kunming 650091, China; 3 Department of Mechanical, Materials and Aerospace Engineering, Illinois Institute of Technology, Chicago, USA

## Abstract

Developing an innovative “Test Paper” based on virgin nanoporous silicon (NPSi) which shows intense visible emission and excellent fluorescence stability. The visual fluorescence quenching “Test Paper” was highly selective and sensitive recognizing Cu^2+^ at μmol/L level. Within the concentration range of 5 × 10^−7^ ~50 × 10^−7^mol/L, the linear regression equation of I_PL_ = 1226.3-13.6[C_Cu_^2+^] (R = 0.99) was established for Cu^2+^ quantitative detection. And finally, Cu^2+^ fluorescence quenching mechanism of NPSi prober was proposed by studying the surface chemistry change of NPSi and metal ions immersed-NPSi using XPS characterization. The results indicate that SiH_*x*_ species obviously contribute to the PL emission of NPSi, and the introduce of oxidization state and the nonradiative recombination center are responsible for the PL quenching. These results demonstrate how virgin NPSi wafer can serve as Cu^2+^ sensor. This work is of great significant to promote the development of simple instruments that could realize rapid, visible and real-time detection of various toxic metal ions.

Heavy metal ions pollution is one of the most serious environmental problems of this age, threatening global sustainability^1^. The risk of such pollutants, even at “invisible” trace levels, is that they can be progressively concentrated through the food chain and form a threat to human health^2^,^3^. Among heavy metals, despite its less-significant toxicity, copper has become a widely distributed pollutant in natural water as a result of the dumping of electronic trash and mining residues^4^. Long-term exposure to excess copper ions (Cu^2+^) is highly toxic to organisms and the human body^5^,^6^. Thereby, numerous methods have been developed for Cu^2+^ detection, such as inductively coupled plasma mass spectroscopy (ICPMS)^7^, atomic absorption/emission spectroscopy (AAS/AES)^8^, electrochemical detection^9^, and nanomaterial-based probes^10^,^11^. These proposed methods have distinct advantages in certain situations, but there are still some limitations, including expensive equipment, complicated operation, or tedious and time-consuming functionalization process, etc. Therefore, it is urgently needed to develop new methods that could realize rapid, visible and real-time detection of Cu^2+^ with simple instruments^12^,^13^.

Nanoporous Si (NPSi) has been recognized as a versatile material due to its unique physical and chemical characteristics, such as large surface area, strong fluorescence, tunable porous nanostructure and surface chemistry, biocompatibility, which has widely application in photoelectron devices^14^, chemical and biological sensor^15^,^16^, micromachining^17^,^18^, biomedicine^19^ or energy conversion and storage^20^,^21^, etc. To design efficient metal ion sensors, several innovative recognizing groups have been designed and immobilized on NPSi surface. Sam *et al*.^22^ covalently anchored a Glycyl-Histidyl-Glycyl-Histidine peptide to the PSi surface using a multi-step reaction scheme, and which showed voltammetric sensing for copper ions in solution. In our previous work, a novel voltammetric sensor based on 3-aminopropyltriethoxysilanes (APTES) functionalized porous silicon was elaborated for sensing Ag^+^ in aqueous solution^23^. Segal *et al*.^24^ have designed and fabricated optical biosensing platform based on horseradish peroxidase (HRP) and laccase immobilized PSi nanostructures, it allows for real-time monitoring of heavy metal ions by enzymatic activity inhibition. Xia *et al*.^25^ proposed that the microwave-induced 10-undecenoic acid modified porous silicon nanoparticles (PSiNPs), which showed excellent fluorescence stability under physiological conditions and highly sensitive to the concentration of Cu^2+^ at mmol/L level. Palestino *et al*.^26^ synthesized the rhodamineorganosilane derivative (Rh-UTES) by one-pot method and then was covalently immobilized on a porous silicon microcavity via triethoxysilane groups and the Rh-UTES hybrid PSi sensor provided an optical qualitative test for Hg^2+^ detection. However, until now, no study about solid virgin NPSi as fluorescence sensor for the detection of Cu^2+^ had been reported. Based on virgin NPSi with intense and stable luminescent emission, in the present work, we have proposed a solid sensor that can monitor Cu^2+^ with the naked-eye by fluorescence quenching. The effect of surface morphology on chemistry structure and luminescent stability of NPSi also have been investigated. The Cu^2+^ sensing mechanism was researched based on the evolution of NPSi surface chemistry.

## Results and Discussions

**Figure 1:**
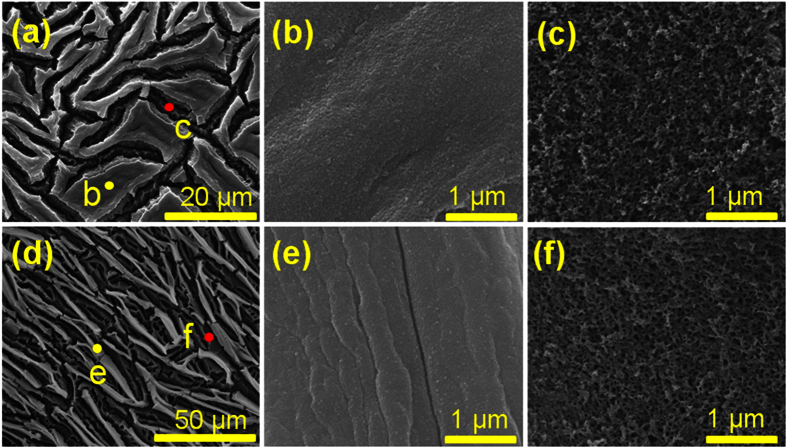
SEM images of the respective NPSi sample I and sample II. (**a**,**d**) images show the overall appearance of sample I and sample II. (**b,c**) images respectively further characterize the constringent cracked layer and porous layer of sample I, which have been marked by yellow and red points in (**a**). (**e,f**) images respectively further characterize the cracked layer and porous layer of sample II, which have been marked by yellow and red points in (**d**).

After the etching, the color of samples show uniform greenyellow and becomes gradually shallow with the decreasing HF concentration. The SEM images show that numerous “ditches” with the depth of ~2 μm are present on entire sample surfaces (Fig. 1a), they can be attributed to the surface shrinkage that caused by the tremendous capillary stress during drying process. The magnified SEM image shows that the edge of the ditch is undulating and relative compact. Crack degrees of the sample surface are dramatically increased. And more numerous “ditches” are observed on the sample surface, almost all surface compact crack layer has fallen off, leading to high exposures of porous layer (Fig. 1b). It indicates that sample II has been suffered with more intense capillary stress and this is further confirmed by the existence of some creases on the edge of the ditch (Fig. 1e). According to the magnified image in Fig. 1f, the pore sizes are slightly increased by comparing with that of Fig. 1c.

**Figure 2:**
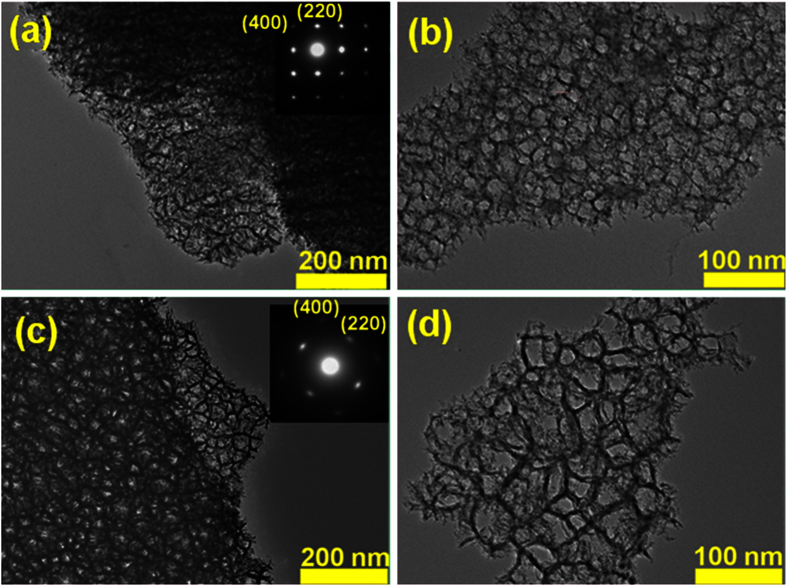
TEM images of sample I (**a,b**) and sample II (**c,d**).

TEM characterization was employed to investigate more detailed morphology and structure of NPSi samples. The typical TEM images and corresponding (001) orientation electron diffraction patterns were shown in Fig. 2. According to Fig. 2, the pores are formed by the knitted skeleton of “silicon wires”, and the “silicon wires” in sample I are denser than that of sample II, which is more resistant to the capillary stress during natural drying process. The corresponding electron diffraction pattern is closely similar to that of characteristic bulk single crystal silicon material, and the diffraction spot of (400) and (220) are assigned in the inset, respectively. There are weak arc-shaped streaks around the main spots, which may be attributed to the presence of disoriented structure in the sample. The explicit pore geometry and diameter distributions can be obtained from the TEM characterization of porous silicon chips. It shows that the irregular polygon structure pores were formed by twisting the silicon nanowires with the diameters of about 4~6 nm, It should be noted that the size of pores are slightly increase with decreasing HF concentration (Fig. 2b,d), which is consistent with previous work^27^.

**Figure 3:**
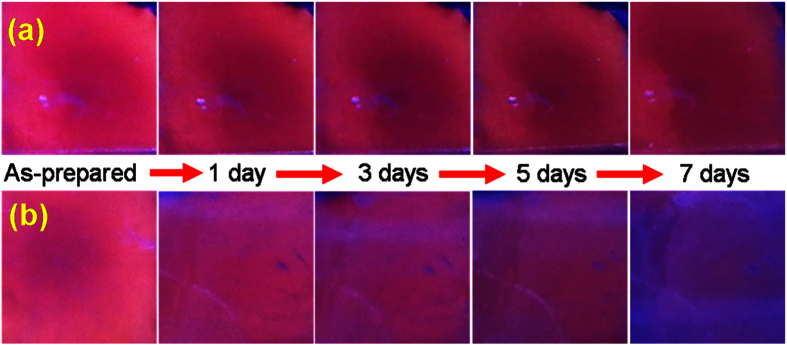
Ultraviolet-excited PL emission photos of NPSi sample under different exposure time in air ambient, (**a,b**) respectively represents sample I and sample II.

Moreover, the fluorescence stability of NPSi was tested in air ambient. The fluorescence emission of NPSi sample was excitated by ultraviolet lamp at the wavelength of 365 nm. Both fresh NPSi glow with the visible orange-red light (Fig. 3), this luminescence activation phenomenon can be explained by quantum confinement effects and defects states^28^. Meanwhile, it can be found that the fluorescence brightness of sample II reduced dramatically with the extending of exposure time, and orange-red light gradually become blue light, because of the instinctive luminescence blue shift behavior of NPSi under air oxidization^25^,^29^. However, we have noted that the fluorescence property of sample I exhibits an excellent stability after seven days aging, its fluorescence brightness is slightly decreased and which is of great importance to the application of visual sensors. Combining with the previous SEM characterization, it is safe to conclude that the existence of numerous constringent layer on sample I surface may slow the oxidation rate of porous layer, and leading to the better luminescence stability.

**Figure 4:**
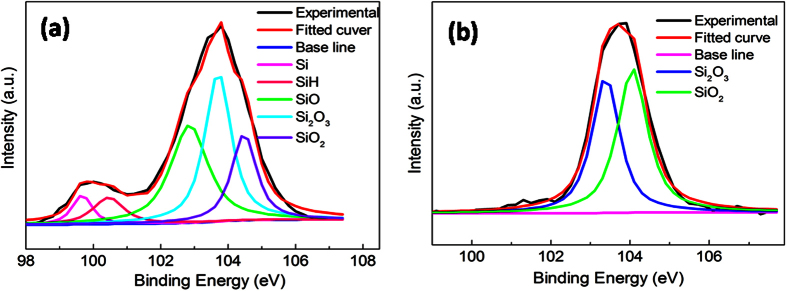
XPS narrow-scans and deconvoluted peaks of Si 2p for NPSi sample after aging in air ambient for 7 days, (**a,b**) respectively represents sample I and sample II.

In order to investigate the effect of surface morphology on surface chemistry of NPSi sample, X-ray photoelectron spectroscopy (XPS) of sample I and II were characterized after aging under air ambient for one week. For sample I, the Si 2p peak can be deconvoluted into four bands at 99.5 eV, 100.4 eV, 102.4 eV, 103.5 eV, and 104.4 eV, which can be assigned to the Si, SiH, SiO, Si_2_O_3_, and SiO_2_ (Fig. 4a). As known, the freshly prepared NPSi surface is mainly covered by numerous SiH_*x*_ (x = 1~3) species, which may be oxidized by O_2_ in ambient^30^, but, residual SiH suggests that NPSi surface has not been fully oxidized. However, the reductive species of SiH_*x*_ completely disappeared according to the deconvoluted Si 2p peak of sample II, which means that the sample surface is totally covered by the oxidizing species of Si_2_O_3_ and SiO_2_ (Fig. 4b). The results confirm that the numerous surface compact layer is benefit for slowing the oxidization process of SiH_*x*_ species, which would be responsible for the better luminescence stability of sample I.

**Figure 5:**
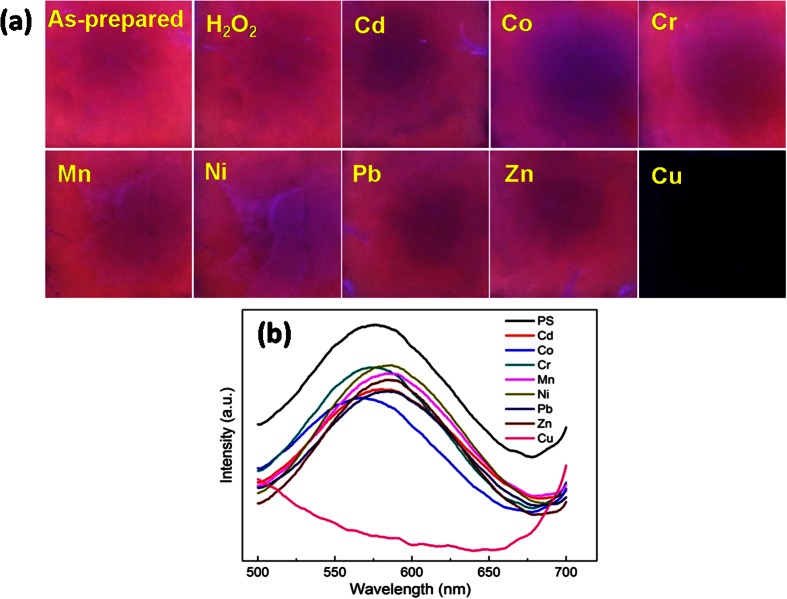
Ultraviolet-excited PL emission photos (**a**) and PL spectrums (**b**) of NPSi after the immersion in solution with different mental ions species.

The resultant sample I with excellent fluorescence stability was selected as fluorescence probes to detect Cu^2+^. Various heavy metal ions solution (Cd^2+^, Co^2+^, Cr^3+^, Mn^2+^, Ni^2+^, Pb^2+^, Ni^2+^, Zn^2+^ as interference species, and Cu^2+^ as target metal ion) were prepared. The concentrations of interference metal ions and target metal ions are respectively 500 μmol/L and 10 μmol/L. To evaluate the selectivity of NPSi sensor, the freshly prepared NPSi sample was immersed into different metal ions solution for 30 min. After that, the samples were removed and dried under nitrogen for fluorescence characterization. The corresponding ultraviolet lamp excited fluorescence photos and the photoluminescence spectra were shown in Fig. 5. Remarkably, there was a significant fluorescence quenching of NPSi with the only addition of Cu^2+^. Due to the capture of excited carriers and the interruption of the radiative recombination, Cu^2+^ could dramatically reduce the quantum efficiency of NPSi^31^. Meanwhile, the measured PL spectra characterization shows that the resulting NPSi has an intense luminescence emission at the band at ~575 nm. For those NPSi samples immersed into solution with interference metal ions, the luminescence intensity decreases slightly ~10% by comparing that of fresh one. Therefore, it is safe to conclude that the NPSi has selectively fluorescence sensing properties for Cu^2+^.

**Figure 6:**
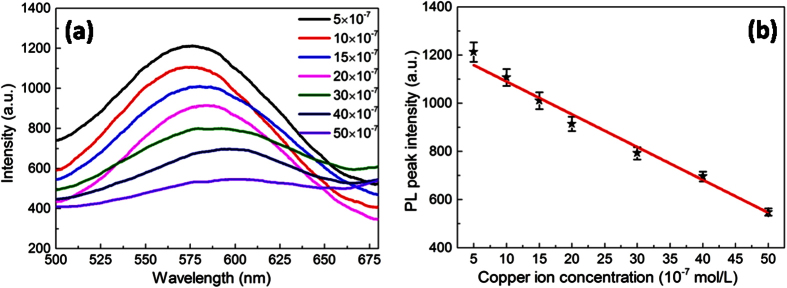
Effect of copper ions concentration on PL intensity of NPSi (**a**) and the relationship between PL peaks intensity and Cu^2+^ concentration (**b**).

Furthermore, the fluorescent sensing of NPSi toward Cu^2+^ was carried out to investigate the working principle. The PL intensity (I_PL_) at ~575 nm shows continuous quenching with the increasing Cu^2+^ concentration (Fig. 6a). The intensity of luminescence had a linear relationship within Cu^2+^ concentration range between 5 × 10^−7^ and 50 × 10^−7^mol/L, and the linear regression equation was I_PL_ = 1226.3 -13.6[C_Cu_^2+^], with a correlation coefficient (R) of 0.99.

**Figure 7:**
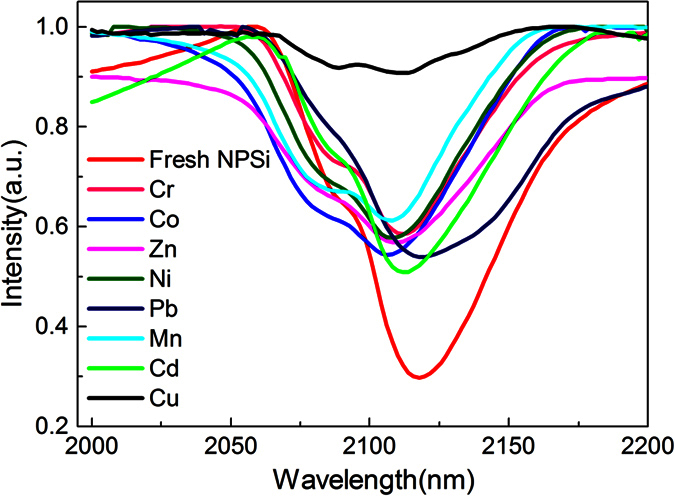


Numerous research has confirmed that the surface chemical species has a crucial effect on luminescence properties of NPSi^31^. In order to illustrate the luminescence quenching mechanism, it is necessary to study the effect of various metal ions immersion on surface chemical composites of NPSi. The SiH*x* groups are the most distinctive species on the fresh NPSi surface and benefit for the intense luminescence emission^32^, but they are sensitive to external environment and affecting luminescence properties of NPSi^33^,^34^. The FTIR spectra of SiH_*x*_ groups of fresh and immersion-treated sample were characterized in Fig. 7. For the freshly prepared NPSi, the visible bands at 2117 and 2089 cm^−1^ are assigned to SiH and SiH_2_ stretching modes, and the signal of SiH3 species at the band of ~2140 cm^−1^ is overlapped in the wide band region^35^. The peak intensity of SiH_*x*_groups decrease when NPSi sample is immersed into aqueous solution, especially for the Cu^2+^ immersed sample it has a dramatic intensity reduction, which may be attributed to the oxidization introduction caused by Cu^2+^ species, and the corresponding reaction can be described as follow. Meanwhile, a slight peak shift can be observed for all of the aqueous solution immersed samples, which can be attributed to the higher polar effect of aqueous solution than that of alcohol.


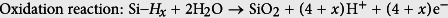



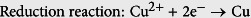


We can also note that the spectra intensity for those NPSi immersed into solution containing various metal ions species all show a slight reduction, and the difference between of them is not evident, thus, we can deduce that the selected interference ions species has no obvious effect on intensity reduce of SiH_*x*_ peak, the slight decay of PL intensity may be mainly caused by the oxidization of H_2_O in the solution.

**Figure 8:**
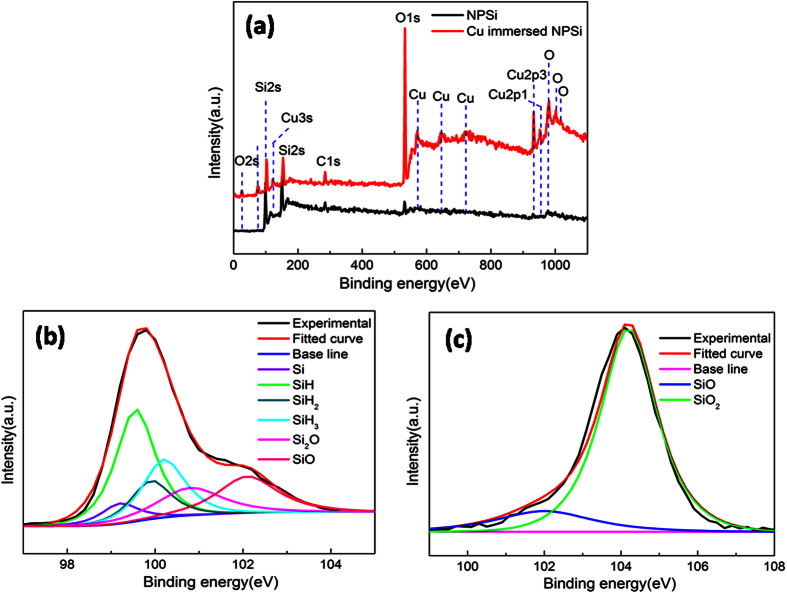
XPS survey-scan (**a**) of un-immersed NPSi and Cu immersed NPSi, Si 2p narrow-scans peaks of un-immersed NPSi (**b**) and Cu immersed NPSi (**c**).

To further confirm the reduction of Cu^2+^ on NPSi surface, the fresh and Cu^2+^-immersed NPSi sample were characterized by XPS spectroscopy, and the results were shown in Fig. 8. The un-immersed NPSi surface mainly contains silicon and a little amount of oxygen (Fig. 8a). After the Cu^2+^-immersion, several new peaks present between the range of 550~730 eV can be assigned to metal copper (Cu) species. It indicates that copper ions are reduced during the immersion step, and leading to the further oxidation of NPSi surface, this is confirmed by the increasing intensity of oxygen peaks. Furthermore, the corresponding Si 2p peaks were deconvoluted and analyzed. For the un-immersed NPSi sample, the Si 2p peak mainly locate at 99.8 eV and has a broadened peak at the higher binding energy, which is attributed to that the bind between Si and H, O. This Si 2p peak can be deconvoluted into six peaks which can be respectively assigned to Si, SiH, SiH_2_, SiH_3_, Si_2_O, and SiO (Fig. 8b). But for Cu^2+^-immersed sample, its surface is totally covered by the oxidizing species of SiO and SiO_2_, there is not any reducing species of SiH_*x*_. When combining the date from Fig. 4, we can notice that the oxidization degree is more serious for Cu^2+^-immersed NPSi sample. Also, it can be concluded that the introduction of oxidization state plays an important role in photoluminescence quenching of NPSi through XPS characterization and luminescence properties. Moreover, Calandra *et al*.^36^ has proved that the deposited Cu can bind with the dangling bonds of silicon and form new surface electronic states near the Fermi energy, which works as nonradiative recombination center, leading to the dramatic decay of PL emission.

**Figure 9:**
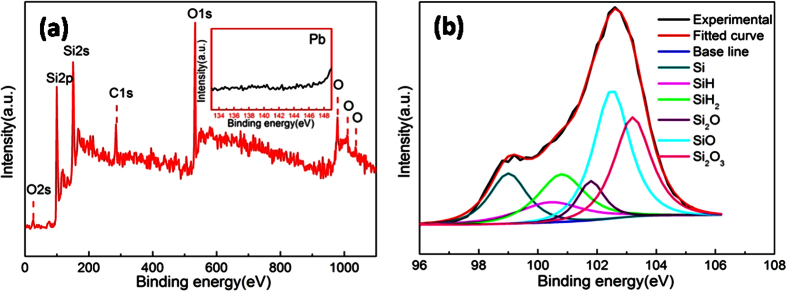
XPS survey-scan spectrum (**a**) and Si 2p narrow-scans peak (**b**) after Pb^2+^ deposition on NPSi.

To reveal the reason of different immersed behavior of various metal ions on NPSi surface, we list they redox potential in Table 1. According to the data in Table 1, all of the selected interference metal ions species have negative redoxpotential except the target Cu^2+^ions. The Cu^2+^ species with positive potential is optimum for charge transfer between NPSi surface and copper ions, which leads to the oxidization of NPSi surface^37^. Among the interference metal ions species, the redox potential of lead ions (Pb^2+^) has the most relatively positive potential, its electroless deposition is the most likely to occur on NPSi surface (Table 1). The Pb^2+^-immersed sample surface was also characterized by XPS. No Pb peak can be found in the survey-scan spectrum, which is further confirmed by the close-up of corresponding binding energy range of lead species in the inset of Fig. 9a. The Pb^2+^-immersed sample surface mainly contains silicon and oxygen species. The Si 2p peak mainly locate around 103 eV and spread on a lower binding energy range (Fig. 9b), and it can be deconvoluted into six peaks which can be respectively assigned to Si, SiH, SiH_2_, Si_2_O, SiO, and Si_2_O_3_. Therefore, Pb^2+^ in solution has no obvious effect on surface chemistry states of NPSi, and thus NPSi sensor has no intense PL decay. It is believed that this result can be naturally generalized to the other interference metal ions since others interference metal ions have more negative redox potential.

## Conclusions

An innovative “test paper” based on the solid flake-like nanoporous silicon has been developed and which shows excellent fluorescence stability. Fluorescence quenching in as-prepared NPSi probe was highly selective and sensitive with copper ions in solution, it exhibits the visual qualitative recognition capability for Cu^2+^ at μmol/L level. The NPSi could be further applied to detect the Cu^2+^ concentration within the range of 5 × 10^−7^~50 × 10^−7^mol/L through the linear regression equation between the PL intensity and the Cu^2+^ions concentration which was firstly established as I_PL_ = 1226.3-13.6[C_Cu_^2+^] (R = 0.99). By comparing the surface chemistry structure evolution of NPSi sample, fluorescence quenching mechanism of NPSi probe was investigated and we concluded that SiH_*x*_ species obviously contribute to the PL emission of NPSi. Formation of oxidization state and the nonradiative recombination center caused by the preferential deposition of Cu^2+^ions are responsible for the PL quenching, and it is mainly attributed to its more positive redox potential of copper ions species.

## Methods

Silicon wafers were purchased from 46th institute of semiconductor of China. P(100)-type single crystal Si wafers with resistivity of 0.01~0.09 Ω·cm were cut into small pieces with a size of ~1 × 1 cm^2^, and then respectively cleaned in ethanol and distilled water for 10 min under sonication. The native oxidation layer was removed by immersing the specimens in 5 wt.% HF aqueous for 20 min. The treated wafers were electrochemically etched in the double-tanks electrochemical cell. The mixture solutions of HF (with concentration of 20 wt.%) and ethanol with volume ratio 1:4 and 1:5 were selected as etchant, etching current density of 25 mA/cm^2^ was imposed by dc power supply, etching time was 60 min, and the resulting samples were respectively marked by sample I and sample II. After etching, the NPSi samples were rinsed with absolute ethanol and dried under dry N_2_ stream. The stock solutions of Cd, Co, Pb, Mn, Zn, Ni, Cr and Cu were prepared by dissolving metal salts in ultrapure water, which contains CdCl_2_, CoCl_2_·6H_2_O, PbCl_2_, MnCl_2_·4H_2_O, MnCl_2_·4H_2_O, NiCl_2_·6H_2_O, CrCl_3_·6H_2_O and CuCl_2_·2H_2_O, they were all purchased from China National Medicines Corporation Ltd. The test processes were implemented by immersing NPSi sensor into 100 ml solution with a fixed metal ions concentrations for 10 min, the immersion solution can be prepared by diluting the stock solution without any pH adjustion. After the test, the NPSi was rinsed with DI water and dried under N_2_ stream.

NPSi layer morphology was characterized by scanning electron microscopy (FEI, QUANTA200). The detail characterization were performed by the JEM-2100 high-resolution transmission electron microscope (HRTEM, JEOL). The samples were prepared by spreading NPSi layer into ethanol and then salvaging with copper grids. The HRTEM characterization was operated at 200 kV. The HRTEM images were analyzed to obtain interplanar spacing (d) and the diffraction pattern. The photoluminescence (PL) spectra of solid NPSi wafer were measured by F-320 fluorescence spectrophotometer at the room temperature, and the excitation wave length was 365 nm. The FTIR characterization was performed by fourier transform infrared spectroscopy (Bruker EQUINOX 55). XPS analysis was performed by the PHI5600 X-ray photoelectron spectroscopy (PHI, United States).

## Additional Information

**How to cite this article**: Li, S. *et al*. An Innovative Metal Ions Sensitive ‘‘Test Paper’’ Based on Virgin Nanoporous Silicon Wafer: Highly Selective to Copper(II). *Sci. Rep.* **6**, 36654; doi: 10.1038/srep36654 (2016).

## Figures and Tables

**Table 1:** Standard redox potential of the selected metal ions and redox potential under different concentration.

Metal ions species	Standard redox potential (V)	Concentration (mol/L)	Redox potential (V)
Cd^2+^/Cd	−0.403	1 × 10^−4^	−0.521
Co^2+^/Co	−0.277	1 × 10^−4^	−0.395
Pb^2+^/Pb	−0.126	1 × 10^−4^	−0.244
Mn^2+^/Mn	−1.17	1 × 10^−4^	−1.29
Zn^2+^/Zn	−0.763	1 × 10^−4^	−0.881
Ni^2+^/Ni	−0.257	1 × 10^−4^	−0.375
Cr^3+^/Cr	−0.740	1 × 10^−4^	−0.858
Cu^2+^/Cu	0.340	1 × 10^−4^	0.222
Cu^2+^/Cu	0.340	50 × 10^−4^	0.272
Cu^2+^/Cu	0.340	1 × 10^−7^	0.133
